# Development and National Implementation of a Standardized Physiotherapy Assessment Package for Pediatric Asthma and Dysfunctional Breathing

**DOI:** 10.1002/ppul.71845

**Published:** 2026-09-22

**Authors:** Charlotte Wells, Claire Hepworth, Nicki Barker

**Affiliations:** ^1^ Suffolk University Ipswich UK; ^2^ Imperial College London London UK; ^3^ Alder Hey Children's NHS Foundation Trust Liverpool UK; ^4^ Sheffield Children's NHS Foundation Trust Sheffield UK

**Keywords:** asthma, dysfunctional breathing, pediatric, physiotherapy

## Abstract

**Introduction:**

Variation in pediatric respiratory physiotherapy assessment for asthma and dysfunctional breathing (DB) across NHS services creates inconsistency in clinical reasoning, workforce development, and data harmonization. We aimed to design and implement a standardized, evidence‐informed assessment package to support physiotherapists working with children and young people (CYP) with asthma and DB.

**Methods:**

Evidence from a previously conducted structured scoping review (PROSPERO ID: 112227) informed key assessment domains. A national collaborative of specialist pediatric respiratory physiotherapists (Kids' Cornerstone Collaborative) co‐developed a standardized assessment proforma, competency framework (Agenda for Change Band 5–8), and supporting educational materials. An iterative, problem‐driven implementation approach was piloted across five NHS sites and refined using a Theory of Change framework. National dissemination occurred via professional networks and conferences. Uptake was assessed through formal access requests and user agreements.

**Results:**

The final package included a standardized pediatric asthma and DB assessment proforma (digital and paper formats), an implementation playbook, competency framework, and educational resources. Intellectual property agreements enabled free NHS national adoption. Fifty‐six NHS Trusts across all UK regions requested access; 30 completed user agreements and received the full package. Pilot implementation informed iterative refinement and strengthened the national collaborative infrastructure.

**Conclusion:**

We developed and nationally disseminated a standardized physiotherapy assessment package for CYP with asthma and/or DB, achieving broad professional engagement across the United KIngdom. This initiative supports consistent evidence‐based clinical practice, structured competency development, and future harmonized outcome evaluation across NHS services.

**Abbreviations**
CSP: Chartered Society of Physiotherapy
CYP: children and young people
DB: dysfunctional breathing
EILO: exercise induced laryngeal obstruction
HCPC: Health Care Professions Council
IP: intellectual property
IT: information technology
NHS: National Health Service
ToC: theory of change
UK: United Kingdom

## 1 Introduction

Pediatric asthma management has evolved considerably over recent decades; however, the role and scope of physiotherapy within pediatric respiratory services has lacked parallel standardization. In a 2022 professional commentary, it was highlighted how core respiratory physiotherapy skills were eroded following shifts in asthma management paradigms during the late twentieth century, with consequent variability in clinical practice and professional identity within pediatric asthma care [1]. That paper called for renewed clarity around the physiotherapist's contribution to respiratory assessment and symptom analysis in children and young people (CYP).

Subsequent benchmarking of pediatric difficult asthma physiotherapy services demonstrated substantial variation in staffing models, assessment processes, competency expectations, and access to specialist input across NHS services [2]. This heterogeneity potentially creates inconsistency in clinical reasoning, inequity in service provision, and constrains opportunities for harmonized data collection and collaborative research. Variation is particularly evident in the assessment of breathlessness and dysfunctional breathing (DB), where nuanced symptom interpretation is essential for distinguishing asthma pathology from disordered respiratory patterns.

In adult respiratory care, recent consensus work has addressed similar challenges. Grillo et al. [3] established agreed terminology and assessment principles for breathlessness and disordered breathing patterns, providing a structured framework to support physiotherapy assessment and interdisciplinary communication. However, no equivalent nationally agreed assessment framework exists for pediatric asthma and DB. Children present with distinct anatomical, developmental, behavioral, and psychosocial considerations, and pediatric services operate within different workforce and commissioning structures. Direct translation of adult frameworks is therefore insufficient. DB is highlighted as one of the common causes of breathlessness in the Adult Breathlessness guidelines, recommending physiotherapy for breathing control exercises [4], however there are no breathlessness guidelines in pediatric care.

Together, these strands of work identify a clear professional and service‐level gap: the absence of a nationally standardized, evidence‐informed physiotherapy assessment approach for CYP with asthma and DB. Without such standardization, variation in assessment undermines patient care, workforce development, competency progression, service evaluation, and the generation of comparable outcome data across NHS hospitals in the United Kingdom.

DB is recognized nationally and internationally as a significant co‐morbidity in asthma that can mimic and amplify symptoms, making asthma appear treatment resistant [5]. It has a high associated symptom burden, significantly affects quality of life and should be considered as part of the assessment of patients with poorly controlled asthma [6, 7]. Asthma is the most common long‐term condition in pediatrics, and the UK has amongst the highest mortality rates in Europe due to asthma attacks [8]. The CYP Asthma Capability Framework provides explicit competency requirements for recognizing and managing comorbidities including DB [9].

The aim of this study was therefore to design and implement a national standardized physiotherapy assessment package, including supporting educational and competency resources, for pediatric asthma and DB services across the NHS. By establishing shared assessment principles and tools, we sought to reduce unwarranted variation in practice, strengthen professional capability, and create infrastructure for future harmonized evaluation of clinical outcomes.

## 2 Methods

### 2.1 Conceptual Framework and Theory of Change (ToC)

**Figure 1:**
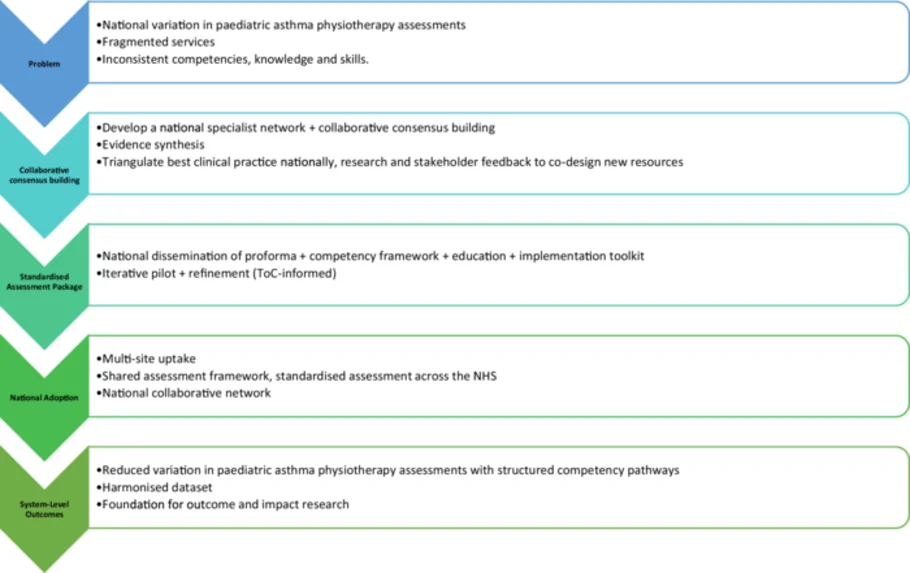
From national practice variation to standardized pediatric respiratory physiotherapy assessment: Development and implementation pathway. [Color figure can be viewed at wileyonlinelibrary.com]

**Figure 2:**
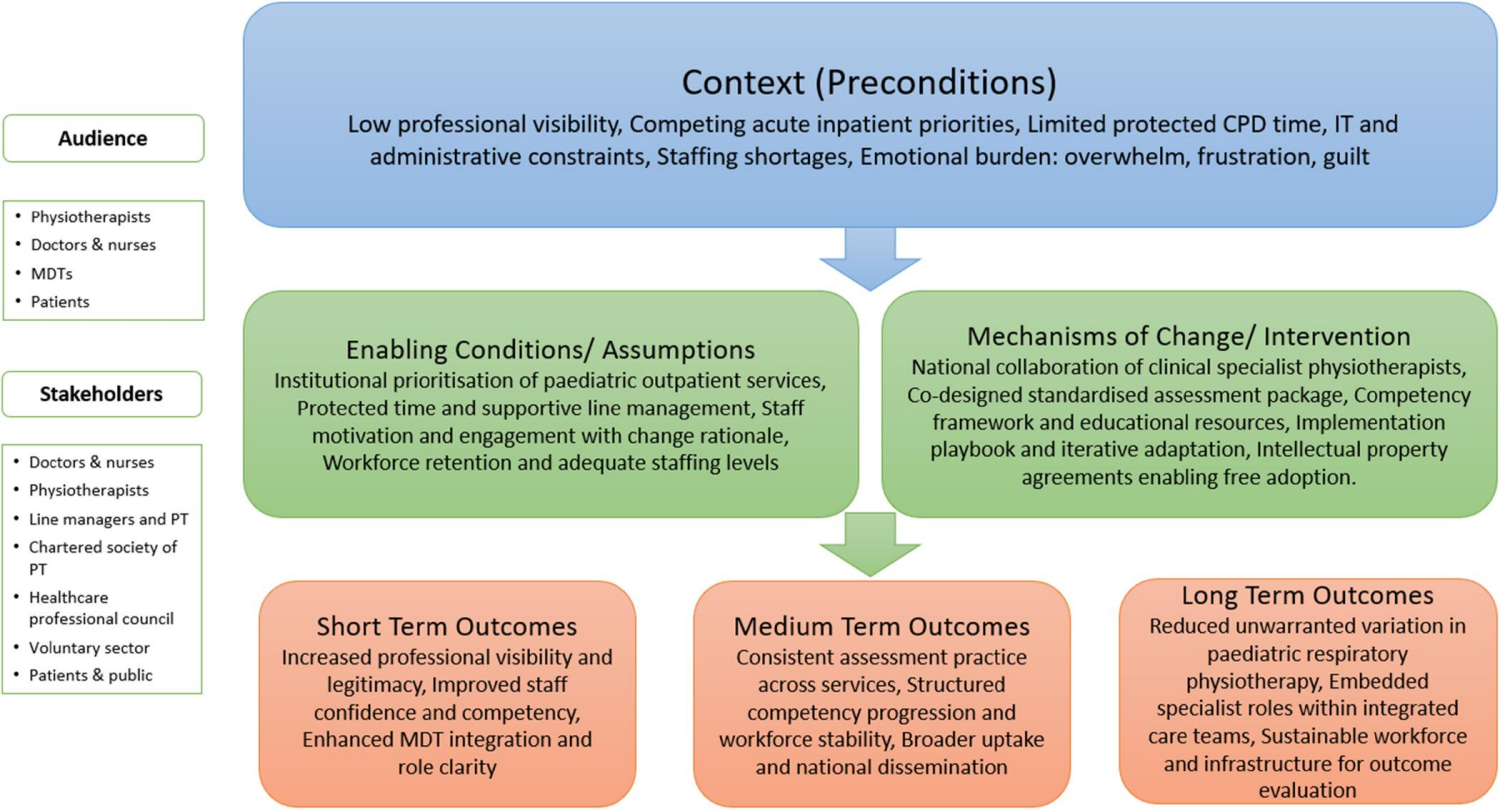
Theory of change underpinning the national co‐design and implementation of a standardized pediatric respiratory physiotherapy assessment package. The model illustrates the progression from identified national variation in assessment practice to system‐level standardization. Collaborative specialist leadership, evidence synthesis, and iterative implementation processes generated a standardized assessment package supported by competency and educational resources. Short‐ and medium‐term outcomes include national adoption, strengthened workforce capability, and service‐level integration. Long‐term change aims to reduce unwarranted variation and establish infrastructure for harmonized data collection and future effectiveness research. [Color figure can be viewed at wileyonlinelibrary.com]

Variation in clinical practice in pediatric respiratory physiotherapy, particularly for CYP with asthma and DB, was identified across NHS services [2]. To address this, a conceptual system change pathway was developed linking the problem (variation in practice) to the solution (standardization of competencies, assessment, and education). This strategy mapped key drivers for change, including knowledge gaps, inconsistent assessment approaches, and variability in interventions (Figure 1). This project was underpinned by a ToC framework, a planning and evaluation approach that maps how activities are expected to lead to desired outcomes and system change [10]. The ToC conceptualizes the process from identified variation in pediatric respiratory physiotherapy practices to national standardization through collaborative design, workforce development, and service embedding. The ToC was developed through workshops with frontline physiotherapists and service users, incorporating contextual and institutional barriers such as workforce capacity constraints, limited protected time, IT and administrative challenges, and emotional burden [11]. This identified enabling conditions necessary for successful implementation for innovations within this clinical area (e.g., institutional prioritization, supportive line management, workforce retention) (Figure 2).

### 2.2 Literature Review and Evidence Synthesis

**Figure 3:**
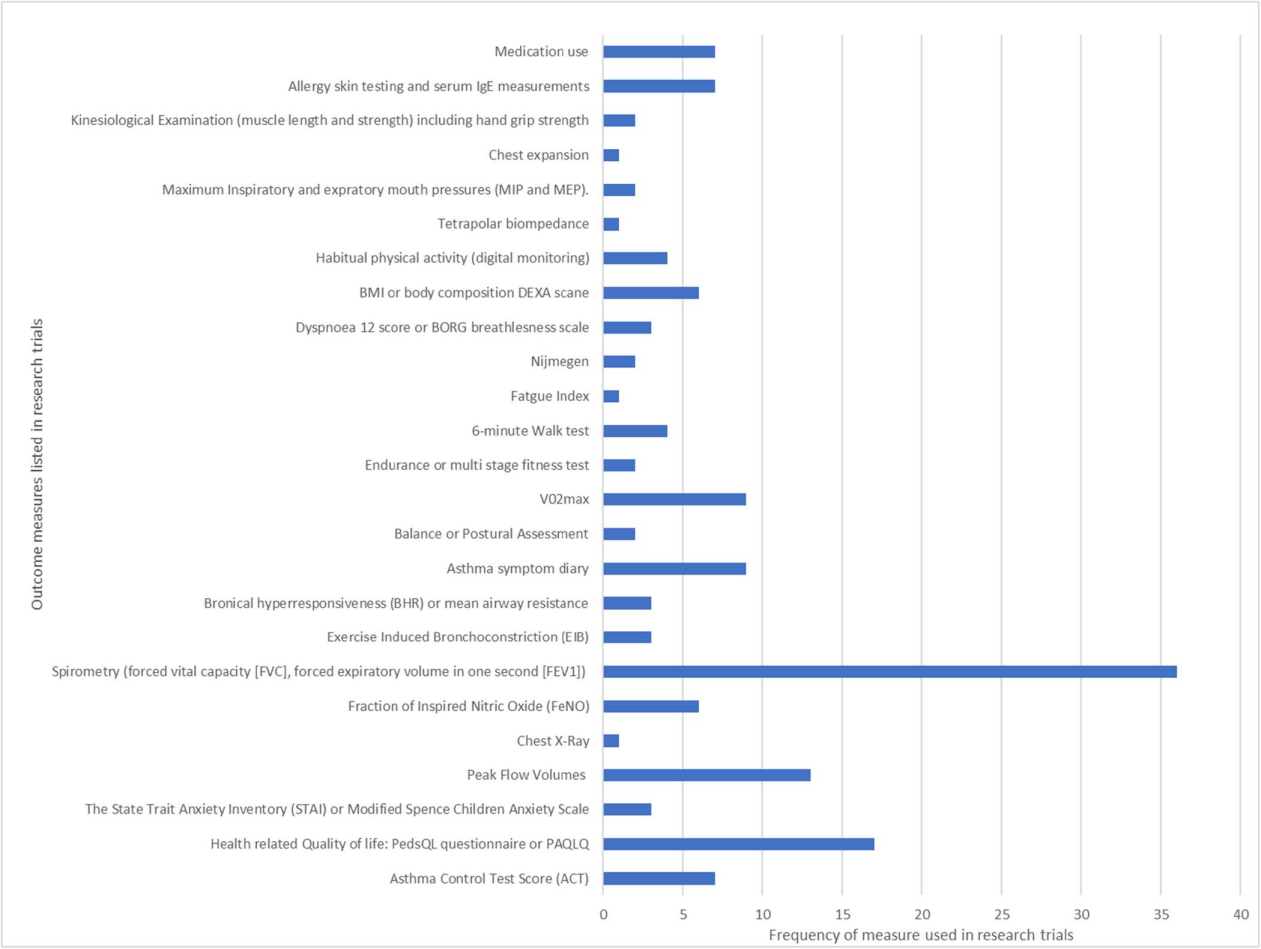
Outcome measure usage across studies identified in Structure Scoping review. [Color figure can be viewed at wileyonlinelibrary.com]

Evidence from a previously conducted structured scoping review (PROSPERO registration ID: 112227; manuscript in preparation) was used to inform the development of the assessment framework (see Supplementary Information S1 for details). The review identified relevant non‐pharmacological physiotherapy interventions for pediatric asthma and DB. An element of this review focused on assessment approaches and outcome measures reported in the literature, which contributed to the development of a physiotherapy‐specific standardized assessment framework (Figure 3). Findings from this evidence synthesis were used to inform and triangulate with clinical expertise and current best practice during the development of the standardized assessment package, competency framework, and educational resources.

### 2.3 National Clinical Specialist Working Group

A national working group of clinical specialist physiotherapists in pediatric respiratory care was established, encompassing geographical representatives from across the United Kingdom (Sheffield, London, Cambridge, and Liverpool) comprising one Band 8b, two Band 8a, and two Band 7 physiotherapists, chosen for their expertise in severe asthma services (Tier 4/5) and/or DB, including exercise‐induced laryngeal obstruction (EILO) and teaching in higher education. Over a 12‐month period, the group convened in four structured meetings of approximately 2.5 h each. The group employed a standardized approach to decision‐making that facilitated inclusive discussion, proposal formation, and modification. Each meeting followed a clear agenda and ensured equal input from all members. Consensus on decisions, competencies, and assessment tools was achieved through iterative discussion, requiring ≥ 80% agreement. This process facilitated refinement of the assessment framework, and competencies in response to emerging clinical and educational insights.

Individual physiotherapists were tasked with leading the development of specific assessment sections and corresponding educational materials designed to scaffold skill and knowledge acquisition during clinical use of the assessment. Between meetings, members provided iterative feedback via comments and preferences, which were collated and circulated to ensure transparency and collective refinement.

### 2.4 Development and Iterative Implementation

The working group collaboratively agreed on the final content of the standardized assessment, which was professionally designed into an interactive and basic print PDF format, interactive word document, and Microsoft Forms version. This assessment package was initially piloted across five NHS sites represented in the working group.

Using a problem‐driven iterative adaptation approach [12], the implementation strategy was continuously refined to address barriers and contextual variations across sites. Each competency and associated educational resource were trialed and refined based on feedback from staff and supervisors. Supervisors validated competency acquisition through observation, case discussions, and reflective practice. This iterative cycle reinforced the ToC by demonstrating practical pathways from standardized education to consistent clinical practice and measurable outcomes.

### 2.5 Intellectual Property (IP) and Licensing

IP agreements were negotiated across three inventor NHS sites to enable free access and use of the resources by NHS physiotherapists. A formal IP licence was developed through the lead Trust legal team and circulated to the two additional Trust's research and legal teams. The licence underwent iterative refinement over 12 months before final approval, ensuring appropriate governance for sharing and use of the materials.

In parallel, a user agreement was established for staff within the inventor sites to ensure that use and ongoing development of the materials were coordinated through a central governance process, thereby maintaining consistency and integrity of the assessment package.

## 3 Results

### 3.1 Competency and Education Package Development

An evidence‐based physiotherapy assessment and competency package was developed to standardize care for CYP with asthma and DB across NHS services, while supporting progressive staff development against knowledge and skills frameworks in this clinical area. The package is designed not only as a set of resources, but as a practice‐based learning tool, whereby repeated use of the assessment supports the ongoing development of physiotherapists' clinical knowledge, reasoning, and skills, consistent with experimental and reflective learning theories, where knowledge is developed through repeated clinical application and reflection on practice [13, 14, 15].

The package comprises:

- Implementation playbook to support local embedding of the assessment in clinical services.
- Pediatric asthma and DB interactive assessment proforma, available in Word, PDF, and Microsoft Forms formats.
- Supplementary educational document, available in Word and Microsoft SWAY formats.
- Competency framework guiding physiotherapist development across NHS Bands 5–8. Three core competency domains, general respiratory physiotherapy, pediatric asthma, and DB, are provided for Bands 6–8b, with an optional advanced set for highly specialist staff (Bands 7–8a). Learning objectives were aligned with Bloom's taxonomy.

Over 20 educational resources, including online modules, webinars, videos, and service‐specific materials, support competency development, clinical assessment, and patient/family education. The package aligns with national care bundles and Health and Care Professions Council (HCPC) professional standards.

The assessment package was promoted through a workshop at a national pediatric respiratory conference and a professional network group “Lunch & Learn” session. Fifty‐six NHS sites requested access, with thirty granted temporary licenses while formal IP agreements were processed. One site has completed the formal IP licence agreement to date. Staff engagement spanned Bands 6–8b, representing Tier 4 and Tier 5 severe asthma services [9]. Feedback indicated the package was perceived as clear, practical, and immediately applicable in clinical practice.

### 3.2 Problem Driven Iterative Adaptations

1. Intellectual property licencing
  Formal IP licence agreements were developed through the lead Trust's legal team and iteratively refined with two additional research/legal teams over 12 months. Due to delays from low prioritization and complexity in identifying appropriate departmental signatories, a temporary licence system was implemented. This process could be completed by the physiotherapist requesting the assessment package allowing immediate access while formal agreements were processed.
2. Digital solutions

To support adoption, a complementary implementation playbook was developed to facilitate consistent use and integration into local clinical workflows. Key adaptations included creating multiple versions of the assessment document to accommodate diverse NHS IT systems and mitigate IT compatibility challenges. This theoretically allowed basic solutions for every NHS service to enhance access and use of the assessment form, however, some services still faced challenges to adoption where the practical support from IT services was not forthcoming.

### 3.3 Implementation and Early Uptake

- Thirty NHS sites received temporary licenses, with physiotherapists across Bands 6–8b engaging with the materials (Table 1).
- Early feedback indicated improved confidence and consistency in assessment and treatment planning.
- Multiple versions of the package supported integration into most local electronic health records and paper‐based workflows.
- Iterative refinements continue to be informed by ongoing clinical use, feedback, and emerging evidence.

**Table 1:** Summary of development and implementation stages and outcomes.

Stage	Key activities	Working group/Participants	Outputs/Adaptations	Early uptake/Metrics
Problem identification	Variation in physiotherapy practice for CYP with asthma and dysfunctional breathing; lack of standardized assessment	Core team: 1 Band 8b, 2 Band 8a, 2 Band 7 physiotherapists (Tier 4/5 and dysfunctional breathing expertise)	Conceptual pathway linking variation → standardization	—
Theory of change development	Mapping problem → solution; identifying key competencies; aligning with national standards	Working group meetings; iterative discussion	Refined model of change; iterative adjustments after each meeting	—
Literature review	Identifying validated outcome measures to demonstrate clinical change pre/post physiotherapy; supplementing gaps with clinical best practice	Working group	Evidence‐informed selection of assessment tools and educational content	—
Competency and assessment package development	Drafting assessment proforma, supplementary education documents, competency framework; aligning with Bloom's taxonomy	Working group	Package includes: • Interactive assessment (Word, PDF, Forms) • Educational documents (Word, SWAY) • Competency framework (Bands 6–8b, optional advanced B7–8a) • 20+ supporting educational resources	—
IP licensing and digital adaptation	Formal IP agreement developed over 12 months; temporary licence system implemented; multiple formats created for NHS IT systems	Lead trust legal team + 2 research/legal teams	Temporary licence enabled immediate access; multiple digital formats; implementation playbook	30 NHS sites accessed package via temporary licence
Iterative problem‐driven adaptation (PDIA)	Feedback integration from early users and workshops; quarterly working group review; consensus ≥ 80%	Working group + early adopters	Adjusted assessment and education materials; usability improvements; competency refinement	56 NHS sites requested access; early positive feedback on usability and consistency
Dissemination and implementation	Workshop at national conference; Lunch and learn teaching; guidance for embedding into clinical workflows	Developers + wider clinical network	Awareness raised; guidance for local embedding	30 sites using temporary licenses; physiotherapists across Bands 6–8b engaging with materials

## 4 Discussion

This project has developed a comprehensive, evidence‐based physiotherapy assessment, education, and competency package for CYP with asthma and DB, addressing significant variation in clinical practice across NHS sites. Variation in assessment approaches, treatment techniques, and competency expectations has been previously highlighted as a barrier to consistent, high‐quality care in pediatric respiratory physiotherapy [1, 2]. By standardizing competencies, assessment tools, and educational resources, this initiative provides a structured framework to support physiotherapists from NHS Bands 5–8, with optional advanced competencies for highly specialist clinicians (Bands 7–8a), promoting consistent, evidence‐informed practice. This is in line with evidence that competency frameworks enhance workforce development, quality, and consistency of care [16].

A key strength of the project was the integration of research evidence and expert clinical consensus. The literature review specifically identified outcome measures used in research to demonstrate pre‐ and post‐intervention change in CYP with asthma and DB. Where evidence was insufficient, clinical best practice informed the development of assessment items and educational content. This dual approach ensured that the package is both evidence‐aligned and pragmatic, reflecting the realities of pediatric respiratory physiotherapy services, consistent with evidence‐based practice principles that integrate research evidence with clinical expertise and consensus to inform practical decision‐making [17, 18]. Importantly, the working group comprised physiotherapists with NHS Bands 7–8b expertise, experienced in severe asthma services (Tier 4–5) and DB including EILO. Consensus was achieved through iterative discussion, with over 80% agreement required for inclusion of each competency, strengthening the rigor of the framework [19].

The development process was guided by a problem‐driven iterative adaptation approach, allowing continuous refinement of both the ToC and the practical tools [20]. The initial conceptual pathway identified variation in practice as the problem and standardization through structured competencies and assessment as the solution. Subsequent iterative cycles refined the ToC, integrating stakeholder feedback, usability testing, and digital adaptations to maximize the feasibility of implementation across diverse NHS sites. Adaptations included multiple digital formats (Word, PDF, Microsoft Forms, and SWAY) and an implementation playbook, facilitating uptake in services with differing IT infrastructure and clinical workflows.

IP licensing presented practical challenges, reflecting real‐world barriers to national dissemination. Delays in formal approvals were mitigated by establishing a temporary licence system, allowing 30 sites to access the package while formal agreements were processed. This approach demonstrates how pragmatic solutions can support early adoption without compromising governance or IP protection [21, 22]. Future work should explore strategies to streamline licensing processes, particularly for resources with national relevance and educational impact.

Early dissemination and adoption were encouraging. Fifty‐six NHS sites requested access to the package following workshops at national pediatric respiratory conferences and clinical interest group teaching events. While only one site has completed the formal IP licence at the time of reporting, the temporary licence system enabled immediate engagement, highlighting strong demand for standardized resources and educational support in this clinical area. The integration of over 20 educational resources, aligned with Bloom's taxonomy [23] and professional standards outlined by the HCPC [24], ensured that learning objectives and materials were both educationally robust and consistent with recognized professional expectations. Professional standards, provide a structured pathway for physiotherapists to progress across NHS bands and support ongoing continuous professional development and competency maintenance. The package remains available to new users by request via email to kidscornerstonecollaborative@outlook.com.

## 5 Limitations

The package has not yet been formally evaluated for clinical impact or outcomes, and the reliance on expert consensus in areas lacking published evidence introduces potential bias. Additionally, uptake may be influenced by local service priorities, IT infrastructure, and availability of senior mentorship. Future work should include evaluation of clinical outcomes, competency retention, and patient/family experience, alongside consideration of scalability to other pediatric respiratory or chronic conditions.

## 6 Conclusion

In conclusion, this project demonstrates a model for developing and implementing national physiotherapy assessment and competency frameworks, combining evidence, expert consensus, and iterative adaptation. The approach provides a blueprint for addressing variation in practice, supporting workforce development, and improving the quality of care for CYP with asthma and DB. Lessons from IP management, digital accessibility, and cross‐band collaboration are likely to be relevant for future initiatives aiming to standardize physiotherapy practice at scale.

## Author Contributions

**Charlotte Wells:** conceptualization, writing – original draft, methodology, writing – review and editing, project administration, resources, formal analysis, investigation, data curation. **Claire Hepworth:** conceptualization, writing – review and editing, investigation. **Nicki Barker:** conceptualization, writing – review and editing, supervision, investigation.

## Funding

The authors have nothing to report.

## Ethics Statement

The development of the assessment package was a service innovations and educational project and did not involve patient data collection or clinical interventions. As such it did not require formal ethical approval. Participating sites signed a temporary IP licence, providing informed agreement to use the assessment package and contribute to its ongoing development. Professional standards for confidentiality and participation were followed.

## Conflicts of Interest

The authors declare no conflicts of interest.

## Supporting information


Supporting File

## Data Availability

Data sharing is not applicable to this article as no datasets were generated or analyzed during the current study.
