# Supplementary material for: Development and National Implementation of a Standardized Physiotherapy Assessment Package for Pediatric Asthma and Dysfunctional Breathing
Source: Pediatr Pulmonol. 2026 Sep 22;61(9):e71845. doi: 10.1002/ppul.71845 (PMC13595356; doi:10.1002/ppul.71845)
Supplement: Supplementary file 1 [file PPUL-61-0-s001.docx]

**Supplementary Information:**

*Database search terms*

*Literature review search of outcome measures used in physiotherapy research for children with asthma.*

| Article | Article title | Outcome measures used in research trial |
| --- | --- | --- |
| Bignal, 2015 | Breathing retraining for African-American adolescents with asthma: a pilot study of a school-based randomized controlled trial | ACT score, PedsQL questionnaire, Anxiety was measured using The State Trait Anxiety Inventory (STAI), peak flow volumes and spirometry (forced vital capacity [FVC], forced expiratory volume in one second [FEV1]) |
| Kovacikova, 2018 | The effect of balance training intervention on postural stability in children with asthma. | Balance assessment and spirometry measures (forced vital capacity [FVC], forced expiratory volume in one second [FEV1]). |
| Carew, 2018 | Laps or lengths? The effects of different exercise programs on asthma control in children. | Spirometry (forced vital capacity [FVC], forced expiratory volume in one second [FEV1]), peak flow volumes and asthma diary. |
| K. Abde, 2018 Jun 15 | Evaluating pulmonary function, aerobic capacity, and pediatric quality of life following a 10-week aerobic exercise training in school-aged asthmatics: a randomized controlled trial | Spirometry (forced vital capacity [FVC], forced expiratory volume in one second [FEV1]), aerobic capacity (VO2max, 6-minute walk test [6MWT], and fatigue index), and PQoL questionnaire. |
| Wells, 2017 | Benchmarking of paediatric difficult asthma physiotherapy services. | Assessed which OM are being used; objective history taking, subjective assessments, Nijmegen questionnaire, ACT score, D12 questionnaire, quality of life measures, exercise testing maximal and submaximal and spirometry results. |
| Wells, 2019 | Physiotherapy in children with difficult asthma: An exploratory cross-sectional survey of UK services. | Assessed which OM are being used; objective history taking, subjective assessments, Nijmegen questionnaire, ACT score, D12 questionnaire, quality of life measures, exercise testing maximal and submaximal and spirometry results. |
| T.; Fegran, L.; Nilsen, 6 | Active play exercise intervention in children with asthma: A PILOT STUDY | BMI, spirometry (forced vital capacity [FVC], forced expiratory volume in one second [FEV1]), VO2max, PAQLQ, ACT score, and habitual physical activity using a wristband monitor. |
| Yin, 2019 | Appropriate physical training helps to relieve clinical symptoms of pediatric asthma: A meta-analysis. | bronchial hyperresponsiveness (BHR), spirometry (forced vital capacity [FVC], forced expiratory volume in one second FEV1) (FEV1), exercise-induced bronchoconstriction (EIB), and endurance. |
| Gomes, 2013 | Active video game training benefits' in asthmatic children: Pilot study. | FeNO, functional capacity and spirometry (forced vital capacity [FVC], forced expiratory volume in one second [FEV1]). |
| Gomes, 2015 | Active video game exercise training improves the clinical control of asthma in children: Randomized controlled trial | ACT scores, FeNO, maximum exercise testing (CPET), tetrapolar bioimpedance and forced vital capacity [FVC], forced expiratory volume in one second [FEV1]). |
| Sankar, 2018 | Asthma - A Disease of How We Breathe: Role of Breathing Exercises and Pranayam. | Peak flow volumes, spirometry (forced vital capacity [FVC], forced expiratory volume in one second [FEV1]) and quality of life measures. |
| Macedo, 2016 | Breathing exercises for children with asthma (Cochrane review) | Measures of quality of life, asthma symptoms and side effects of treatment. |
| Hughes, 2014 | Childhood asthma and exercise | Symptom reporting. |
| Hepworth, 2016 | An observational study of physiotherapy for children with asthma. | ACT questionnaire scores, Nijmegen questionnaire scores, medication change. |
| Hepworth, 2019 | Assessing the impact of breathing retraining on asthma symptoms and dysfunctional breathing in children. | cACT or ACT scores, Nijmegen Questionnaire scores. |
| Mokina, 2018 | Comparative assessment of the QoL specific parameters in children with bronchial asthma with standard pharmacotherapy, and in additional course of physiotherapy in the pool. | PAQLQ |
| Neumannova, 2011 | Comprehensive spa treatment in the Czech Republic. | Assessment included spirometry (forced vital capacity [FVC], forced expiratory volume in one second [FEV1]), maximal inspiratory (MIP) and expiratory (MEP) mouth pressure examination, chest expansion (CE) and kinesiological examination (muscle strength and length). |
| Xu, 2016 | Effect of massage therapy on pulmonary functions of pediatric asthma: A systematic review and meta-analysis of randomized controlled trials. | Spirometry testing forced vital capacity [FVC], forced expiratory volume in one second [FEV1]. |
| Oreskovic Jemric, 2017 | Effects of specific inspiratory muscle training using an individual training device in children with asthma. | Spirometry (forced vital capacity [FVC], forced expiratory volume in one second [FEV1]) and peak flow volumes |
| Vijayalakshmi, 2015 | Effectiveness of massage therapy on anxiety among asthmatic children-hospital based interventional study. | Anxiety was assessed by using Modified Spence Children Anxiety Scale |
| Winn, 2016 | Effect of high-intensity exercise on aerobic performance and airway inflammation in asthma. | Postural assessment, BMI, multi-stage fitness test and FeNO |
| Crosbie, 2012 | The effect of physical training in children with asthma on pulmonary function, aerobic capacity and health-related quality of life: a systematic review of randomized control trials. | Health related quality of life by use of validated scale, symptoms, spirometry (forced vital capacity [FVC], forced expiratory volume in one second [FEV1]) by use of established diagnostic criteria, VO2peak |
| Chiang, 2009 | Effect of relaxation-breathing training on anxiety and asthma signs/symptoms of children with moderate-to-severe asthma: a randomized controlled trial | Anxiety, self-perceived health status, asthma signs/symptoms, peak flow volumes, and asthma medication use |
| Wang, 2009 | The effects of a swimming intervention for children with asthma | Peak flow volumes and spirometry including FVC, FEV1, FEV1%FVC, FEF50, and (FEF25-75). |
| Wicher, 2010 | Effects of swimming on spirometric parameters and bronchial hyperresponsiveness in children and adolescents with moderate persistent atopic asthma | Spirometry (forced vital capacity [FVC], forced expiratory volume in one second [FEV1]), allergy skin testing, and serum IgE measurement |
| Liu, 2015 | Efficacy of acupuncture in children with asthma: a systematic review | Spirometry (forced vital capacity [FVC], forced expiratory volume in one second [FEV1]), medication use, symptom reporting, and quality of life outcomes |
| Didario, 2009 | Efficacy of chest physiotherapy in pediatric patients with acute asthma exacerbations | Mean changes in airway resistance |
| Mohamed, 2012 | Evaluation of needle acupuncture actions on clinical improvement pulmonary functions, interleukin-6, immunoglobulin-e and medications used of asthmatic children. | Spirometry testing including :VC%, FVC%, FEV1%, FEF25–75%, FEF25%, FEF50%, FEF75%, PEF%, IL-6, IgE, Eosinophilic count. Clinical grading and medications use |
| Wanrooij, 2014 | Exercise training in children with asthma: a systematic review. | Spirometry (forced vital capacity [FVC], forced expiratory volume in one second [FEV1]), cardiorespiratory fitness parameters (CPET), exercise induced bronchoconstriction and quality of life. |
| Lu, 2017 | Four months of a school based exercise intervention improved fitness in normal weight and overweight/obese children with asthma in a minority, low SES population-a pilot study. | Spirometry testing (forced vital capacity [FVC], forced expiratory volume in one second [FEV1]), cardiopulmonary exercise testing (CPET), assessments of habitual physical activity, body composition (DXA scans), computer administered executive function (EF), blood work and questionnaires |
| Lu, 2018 | Four months of a school-based exercise program improved aerobic fitness and clinical outcomes in a Low-SES population of normal weight and overweight/obese children with asthma | Spirometry testing (forced vital capacity [FVC], forced expiratory volume in one second [FEV1]), CPET, assessments of habitual physical activity, body composition (DXA scans), computer administered executive function (EF), blood work sent for complete blood count (CBC) with differential, complete metabolic panel, lipid panel, and Immunoglobulin E., childhood asthma control test or ACT questionnaire, The Children’s Health Survey for Asthma (CHSA), The Pediatric Asthma Quality of Life questionnaire, habitual physical activity and sedentary time were assessed during awake time (8 am to 8 pm) for 7 days |
| Tahan, 2014 | Is yoga training beneficial for exercise induced bronchoconstriction?. | Spirometry (forced vital capacity [FVC], forced expiratory volume in one second [FEV1]), exercise testing CPET, IgE levels, eosinophil counts, skin prick testing |
| Kirkby, 2018 | Benefits of pulmonary rehabilitation in pediatric asthma | BMI, spirometry testing (forced vital capacity [FVC], forced expiratory volume in one second [FEV1]), six-minute walk test (6MWT), BORG breathlessness scale and quality of life questionnaires |
| Wu, 2017 | Massage Therapy in Children with Asthma: A Systematic Review and Meta-Analysis. | Spirometry tests (forced vital capacity [FVC], forced expiratory volume in one second [FEV1]) and peak flow volumes |
| Fattah, 2011 | Pulmonary functions of children with asthma improve following massage therapy. | Spirometry testing (forced vital capacity [FVC], forced expiratory volume in one second [FEV1]) and peak expiratory flow volumes (PEF) |
| Neumannova, 2013 | The effect of pulmonary rehabilitation on health-related quality of life in asthmatic children. | Spirometry testing (forced vital capacity [FVC], forced expiratory volume in one second [FEV1]) |
| El-Akkary, 2013 | Role of leukotrienes in exercise-induced bronchoconstriction before and after a pilot rehabilitation training program | Spirometry testing (forced vital capacity [FVC], forced expiratory volume in one second [FEV1]) |
| Walters, 2013 | Swimming training benefits children and adolescents with asthma: Results of a systematic review. | Spirometry testing (forced vital capacity [FVC], forced expiratory volume in one second [FEV1]) and peak expiratory flow volumes |
| Beggs, 2013 | Swimming training for asthma in children and adolescents aged 18 years and under. | Peak expiratory flow volumes |
| Lin, 2013 | Tai Chi Chuan exercise decreases airway inflammation, improves pulmonary function and quality of life in asthmatic children. | Spirometry tests (forced vital capacity [FVC], forced expiratory volume in one second [FEV1]), and peak expiratory flow (PEF), fractional exhaled nitric oxide (FeNO) level, and the quality of life (PAQLQ) |
| Lin, 2017 | Tai-Chi-Chuan Exercise Improves Pulmonary Function and Decreases Exhaled Nitric Oxide Level in Both Asthmatic and Non-asthmatic Children and Improves Quality of Life in Children with Asthma | Spirometry testing (forced vital capacity [FVC], forced expiratory volume in one second [FEV1]), fractional exhaled nitric oxide (FeNO), and quality of life, as assessed by the Pediatric Asthma Quality of Life Questionnaire (PAQLQ). |
| Andrade, 2014 | The efficacy of aerobic training in improving the inflammatory component of asthmatic children. Randomized trial | Blood testing; plasma cytokines were measured. A score of Habitual Level Physical Activity, respiratory muscle strength and spirometry testing (forced vital capacity [FVC], forced expiratory volume in one second [FEV1]), medication use diary, quality of life was determined using the Pediatric Asthma Quality of Life Questionnaire (PAQLQ) |
| Latorre-Roman, 2014 | The effectiveness of an indoor intermittent training program for improving lung function, physical capacity, body composition and quality of life in children with asthma. | BMI, body density and skin fold measurements. Hand grip strength and leg strength. Six-minute walk test (6MWT) with BORG breathlessness scale and HR monitoring. Spirometry testing (forced vital capacity [FVC], forced expiratory volume in one second [FEV1]) and the PAQLQ (Pediatric Asthma Quality of Life Questionnaire) was used in its Spanish version. |
| Kathiresan, 2011 | Efficacy of home-based pulmonary rehabilitation program on pulmonary functions and quality of life in asthmatic children | Symptom and medication scores quality of life index and spirometry tests (forced vital capacity [FVC], forced expiratory volume in one second [FEV1]) |
| Shanmugananth, 2017 | Effect of osteopathic manipulative technique on peak expiratory flow rate, forced expiratory volume in one second among childhood asthma participants | Spirometry testing (forced vital capacity [FVC], forced expiratory volume in one second [FEV1]) and peak expiratory flow volumes |
| Lu, 2017 | Glucocorticoid receptor expression on circulating leukocytes in healthy and asthmatic adolescents in response to exercise | GR expression in leukocytes was measured using flow cytometry. Peripheral blood mononuclear cell (PBMC) gene expression of GR, GRβ, HSP70, TGFβ1, and TGFβ2 was determined using reverse-transcriptase PCR (RT-PCR). |
| Felicio, 2017 | A randomized crossover study of sputum induction by physiotherapy and hypertonic saline techniques in asthmatic children. | Spirometry (forced vital capacity [FVC], forced expiratory volume in one second [FEV1]) |
| Diemer, 2011 | Vocal cord dysfunction - A rare but important differential diagnosis of asthma in childhood. | X-ray, sweat test and testing for infectious diseases yielded no pathologic results. Pulmonary function tests such as body plethysmography, exhaled nitric oxide and methacholine challenge test without pathological findings. |
